# Supplementary material for: Statin therapy and stroke risk in patients with hypercholesterolaemia: a population-based longitudinal study using electronic health records in South London
Source: BMC Med. 2026 Mar 5;24:231. doi: 10.1186/s12916-026-04752-9 (PMC13072472; doi:10.1186/s12916-026-04752-9)
Supplement: Supplementary file 1 — Additional file 1: Fig. S1 and Table S1. Fig. S1. Calibration plot for the logistic regression modelling statin prescription as a function of covariates. Table S1. Patient characteristics according to stroke record. [file 12916_2026_4752_MOESM1_ESM.pdf]

Statin Therapy and Stroke Risk in Patients with Hypercholesterolaemia: A population-based Longitudinal Study Using Electronic Health Records in South London

Marc Delord[1,*] and Abdel Douiri[1]

[1]School of Life Course & Population Sciences, Department of Population Health Sciences, King's College London, London, United Kingdom
[*]Correspondence: marc.delord@kcl.ac.uk, Tel: +44 20 7848 8710

Supplementary materials

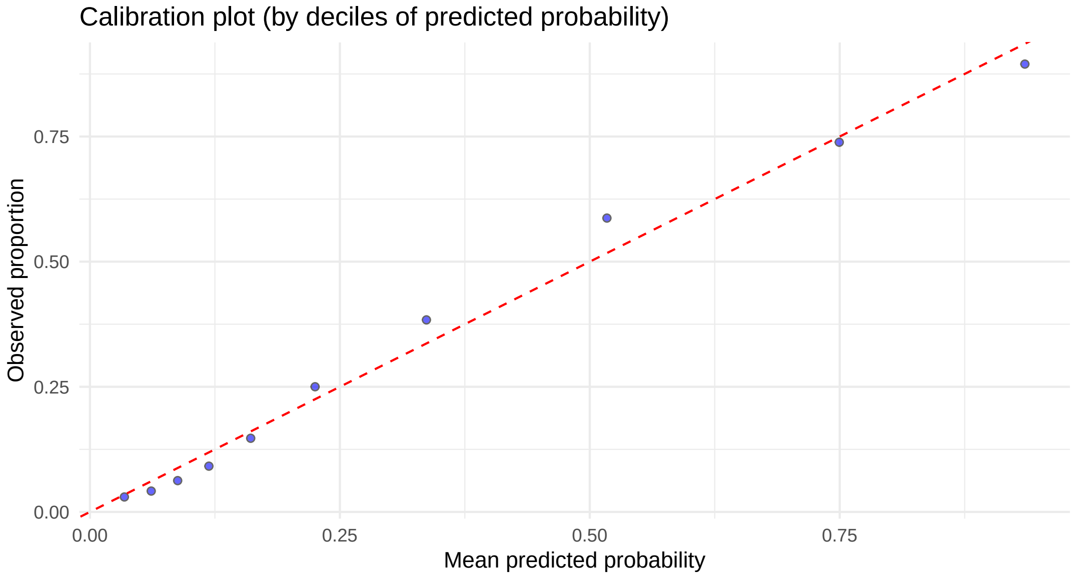

Supplementary Figure S1: *Calibration plot: Calibration for the logistic regression where statin prescription is explain by age, gender, ethnicity, the local quintiles of the index of multiple deprivation, the ever smoking status, hypertension, diabetes, and history of heart diseases.*

Supplementary figure 1 depicts the calibration plot for the logistic regression displayed in figure 2 of the main manuscript. This graph is used to visually asses the agreement between predicted probabilities of statin prescription derived from the fitted model, and observed proportions of patients prescribed statin therapy, by decile of the predicted probabilities. A well-calibrated model should display points lying close to the 45-degree line, indicating good agreement between predicted and observed risks and showing that the logistic regression model fits the data adequately, yielding reliable predicted probabilities.

Supplementary Table S1: *Patient characteristics according to stroke record*

		Stroke		
Patient characteristics		Not recorded	Recorded	Total
Total N (%)		161734 (97.4)	4390 (2.6)	166124
Age at end of follow-up	Median (IQR)	56.1 (22.0)	71.3 (20.5)	56.2 (22.2)
Gender	Male	79923 (49.4)	2194 (50.0)	82117 (49.4)
	Female	81808 (50.6)	2196 (50.0)	84004 (50.6)
Ethnicity	White	88094 (54.5)	2299 (52.4)	90393 (54.4)
	Black	35208 (21.8)	1167 (26.6)	36375 (21.9)
	Asian	13545 (8.4)	326 (7.4)	13871 (8.3)
	Mixed	5997 (3.7)	133 (3.0)	6130 (3.7)
	Other	4597 (2.8)	66 (1.5)	4663 (2.8)
	Unknown	14293 (8.8)	399 (9.1)	14692 (8.8)
IMD local quintile	1 (most deprived)	34680 (21.7)	1137 (26.1)	35817 (21.8)
	2	33548 (21.0)	948 (21.7)	34496 (21.0)
	3	28428 (17.8)	701 (16.1)	29129 (17.7)
	4	29744 (18.6)	683 (15.7)	30427 (18.5)
	5 (least deprived)	33561 (21.0)	892 (20.5)	34453 (21.0)
Age at record of Hypercholesterolaemia	Median (IQR)	46.8 (18.1)	63.1 (19.4)	47.2 (18.4)
Statins	Not prescribed	108813 (67.3)	1709 (38.9)	110522 (66.5)
	Prescribed	52921 (32.7)	2681 (61.1)	55602 (33.5)
Hypertension	No	112064 (69.3)	1427 (32.5)	113491 (68.3)
	Yes	49670 (30.7)	2963 (67.5)	52633 (31.7)
Diabetes	No	136117 (84.2)	3087 (70.3)	139204 (83.8)
	Yes	25617 (15.8)	1303 (29.7)	26920 (16.2)
CKD 3-5[a]	No	150932 (93.3)	3665 (83.5)	154597 (93.1)
	Yes	10802 (6.7)	725 (16.5)	11527 (6.9)
Smoking ever	No	74869 (46.3)	1795 (40.9)	76664 (46.1)
	Yes	86865 (53.7)	2595 (59.1)	89460 (53.9)
Obesity (moderate to severe)	No	104936 (64.9)	2749 (62.6)	107685 (64.8)
	Yes	56798 (35.1)	1641 (37.4)	58439 (35.2)
History of heart disease[b]	No	148282 (91.7)	3109 (70.8)	151391 (91.1)
	Yes	13452 (8.3)	1281 (29.2)	14733 (8.9)
History of cardiovascular disease[c]	No	146113 (90.3)	2536 (57.8)	148649 (89.5)
	Yes	15621 (9.7)	1854 (42.2)	17475 (10.5)
Diabetes*[d] or CKD 3-5	No	131263 (81.2)	2692 (61.3)	133955 (80.6)
	Yes	30471 (18.8)	1698 (38.7)	32169 (19.4)

[a] Chronic Kidney Disease Stage 3-5

[b] Myocardial infarction, Heart Failure, Coronary Heart Disease, Atrial Fibrillation

[c] History of Heart Disease, Transient Ischemic Attack, Peripheral Arterial/Vascular Disease

[d] Diabetes after 40 years old or for more than 10 years
